# MicroRNA-361-3p suppresses tumor cell proliferation and metastasis by directly targeting SH2B1 in NSCLC

**DOI:** 10.1186/s13046-016-0357-4

**Published:** 2016-05-10

**Authors:** Wei Chen, Jun Wang, Sulai Liu, Shaoqiang Wang, Yuanda Cheng, Wolong Zhou, Chaojun Duan, Chunfang Zhang

**Affiliations:** Department of Thoracic Surgery, Xiangya Hospital, Central South University, Xiangya Road 87th, Changsha, 410008 Hunan PR China; Key Laboratory of Cancer Proteomics of Chinese Ministry of Health, Institute of Medical Sciences, Xiangya Hospital, Central South University, Xiangya Road 87th, Changsha, 410008 Hunan PR China; Department of Urology Surgery, Xiangya Hospital, Central South University, Xiangya Road 87th, Changsha, 410008 Hunan PR China

**Keywords:** miR-361-3p, Progression, NSCLC, SH2B1

## Abstract

**Background:**

Lung cancer is the most common malignancies worldwide. However, the detailed molecular mechanisms underlying lung cancer progression are still not completely clear. MicroRNAs are small noncoding RNAs which occupy a crucial role of cancer metastasis. Accumulating evidence suggests that miR-361 plays important roles in human carcinogenesis. However, its precise biological role remains largely elusive, especially in lung cancer. This study examined the role of miR-361-3p in non-small cell lung cancer (NSCLC).

**Methods:**

Real-time quantitative PCR (qRT-PCR) was used to analyze the expression of miR-361-3p in NSCLC tissue and in compared adjacent non-cancerous tissues. The effect of miR-361-3p on proliferation was evaluated by CCK8 and colony formation assays. The effect of miR-361-3p on migration and invasion was evaluated by transwell assays. Western blotting and immunohistochemical staining were applied to analyze the expression of target proteins and downstream molecule, and the luciferase reporter assay to assess the target genes of miR-361-3p in non-small cell lung cancer cells.

**Results:**

miR-361-3p was significantly decreased in NSCLC tissue and cell lines, and its expression levels were highly correlated with lymph node metastasis (*P* < 0.01) and TNM stages (*P* < 0.05). Down-regulation of miR-361-3p promoted cell growth, proliferation, colony formation, invasion and migration in vitro, and promoted proliferation and metastasis in vivo (*P* < 0.01); whereas up-regulation of miR-361-3p had the contrary effects. The luciferase reporter assay showed that SH2B1 was a direct target gene of miR-361-3p. Enforced expression of miR-361-3p inhibited the expression of SH2B1 significantly and the restoration of SH2B1 expression reversed the inhibitory effects of miR-361-3p on NSCLC cell proliferation and metastasis.

**Conclusions:**

miR-361-3p functions as a novel tumor suppressor in NSCLC and the anti-oncogenic activity may involve its inhibition of the target gene SH2B1. These findings suggest the possibility for miR-361-3p as a therapeutic target in NSCLC.

## Background

Lung cancer is the highest mortality malignant tumor in China and African Americans [1, 2]. Non-small-cell lung cancer (NSCLC) accounts for about 80 % of all lung cancer cases, including adenocarcinoma and squamous cell carcinoma. Due to lacking obvious symptoms for early diagnosis and highly malignant potential, the 5-year survival rate of NSCLC is still less than 15 % [3]. Recent years, low-dose computed tomography (LDCT) screening is employed in high risk patients to detect early stage disease, while it was shown to reduce mortality its usefulness is limited by high false-positive rates [4, 5]. These issues highlight the urgent need for accurate that can detect early lung cancer with high sensitivity and specificity.

MicroRNAs (miRNAs) are highly conserved small non-coding regulatory RNAs with sizes of 17–25 nucleotides, which are able to regulate gene expression via binding to the 3'-untranslated regions (UTR) of target mRNAs [6], and was first reported by Ambros and Ruvkun in 1993 [7, 8]. Ample evidence shows that altered miRNA expression result in the initiation, promotion, and progression of NSCLC, such as miRNA-21 [9], miRNA-205 [10], miR-1254 and miR-574-5p [11]. Although the importance of miRNAs has attracted much attention in recent years, the pathological relevance and significance of the majority of miRNAs in NSCLC remain unclear. Thus, understanding of the underlying molecular mechanisms of miRNA dysregulation in malignant tumors is critical to intervention of lung cancer.

Recent studies have shown that miR-361 expression was alternant in several cancer types, for example squamous cell carcinoma [12], cervical cancer [13], prostate cancer [14], colorectal and gastric cancer [15], hepatocellular carcinoma [16]. Such researches imply that miR-361 may play important roles in cancer depending on the tumor type. In this study, we aimed to evaluate the possible roles and related target genes of miR-361-3p in tumorigenesis of NSCLC. We found that the expression level of miR-361-3p in NSCLC was significantly lower in NSCLC tissues than in the corresponding normal lung tissues, and inversely associated with advanced stage and lymph node metastasis of NSCLC. Furthermore, enforced miR-361-3p expression inhibited lung cancer cell growth, proliferation, clone formation, migration and invasion in vitro, and tumorigenicity and intrapulmonary metastasis in vivo. In addition, The SH2B1 was identified as a functional target of miR-361-3p. Therefore, down-regulation of miR-361-3p suppresses lung cancer progression and metastasis through regulation of SH2B1.

## Results

### Expression of miR-361-3p is inversely associated with clinicopathologic characteristics and prognosis of NSCLC

**Fig. 1:**
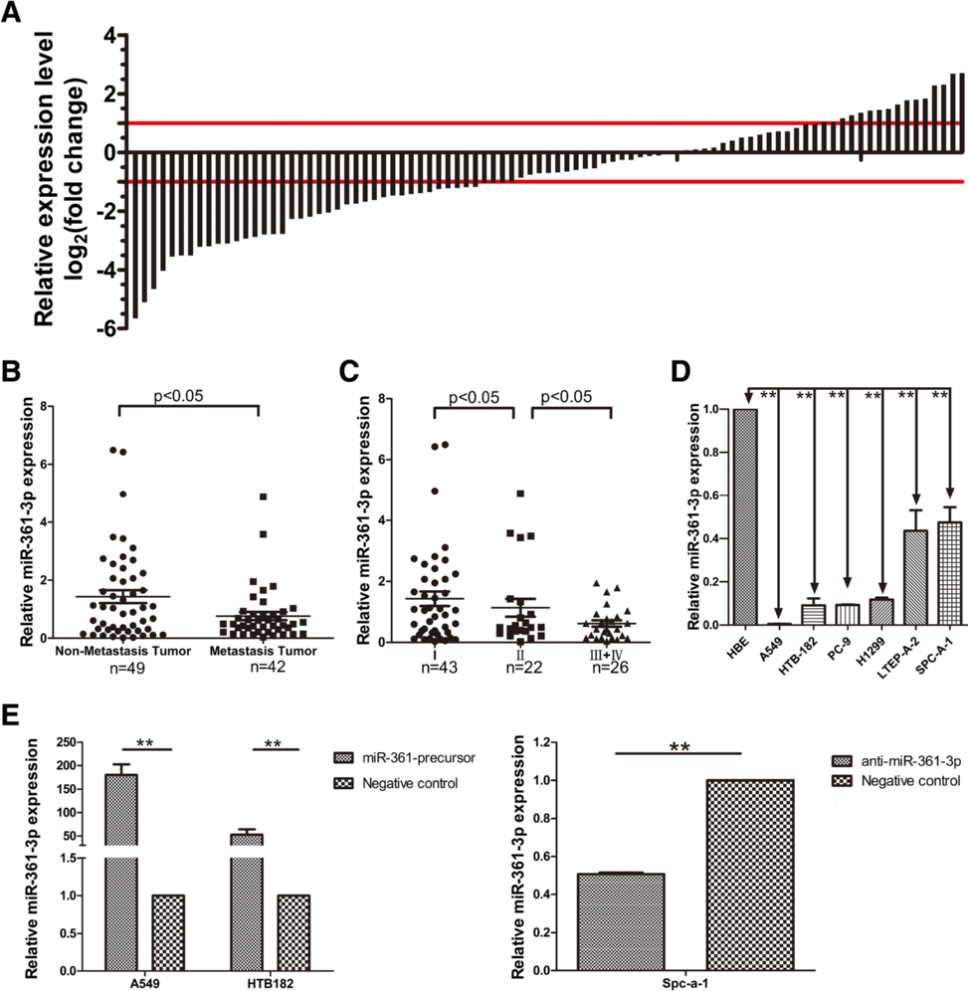
Low expression of miR-361-3p is inversely associated with TNM Classification and lymph node metastasis of NSCLC. **a** The expression of miR-361-3p in 91 pairs of NSCLC tissues was significantly lower. **b** Low-level expression of miR-361-3p was associated with lymph node metastasis of NSCLC, **c** TNM Classification of NSCLC. **d** miR-361-3p expression in NSCLC cell lines and HBE. **e** The level of miR-361-3p was significantly changed in NSCLC cells after infected with miR-361-3p lentivirus or anti-miR-361-3p. Data were represented as the mean ± SEM of three indepedent experiments. **P <0.01

In previous research, we noticed that miR-361-3p was lowexpressed in NSCLC [17]. To confirm, we evaluated the expression of miR-361-3p in 91 pairs of frozen NSCLC tissues and the corresponding normal lung tissues which located 5 cm apart from tumor by quantitative reverse transcriptase PCR (qRT-PCR). For training cohort, miR-361-3p expression was downregulated in NSCLC tissues compared with the matching normal lung tissues, the median was 0.70vs.1 (Fig. 1a). Furthermore, miR-361-3p expression was significantly inversely associated with metastasis and tumor nodes and Metastasis(TNM) stages of the patients (Table 1, *P* < 0.05). In addition, miR-361-3p expression was significantly lower in NSCLC tissue which displayed lymph node metastasis than did not (Fig. 1b) (*P* <0.05), and decreased statistically with increasing stage of NSCLC (*P* < 0.05) (Fig.1c). Therefore, the low miR-361-3p expression was closely related to the progression and metastasis of NSCLC. We also evaluated miR-361-3p expression in six NSCLC cell lines (A549, HTB-182, PC-9, NCI-H1299, LTEP-A-2, SPC-A-1) and a normal human bronchial epithelial cell line (HBE). The relative expression levels for miR-361-3p in these six NSCLC cell lines were 0.005, 0.091, 0.093, 0.118, 0.436, and 0.475, respectively, as compared with that of HBE cells, respectively (Fig. 1d). Remarkably, A549 a squamous cell carcinoma cell line, and HTB-182 an adenocarcinoma cell line, which expressed the lowest miR-361-3p level, consistent with the association of miR-361-3p with NSCLC metastasis as observed in NSCLC patient samples.

**Table 1:** miR-361-3p expression and clinicopathological features in non-small cell lung cancer (NSCLC) patients

Variables	n	miR-361-3p expression		
		High expression	Low expression	*p* value^*^
Gender				
Male	67	27(40 %)	40(60 %)	
Female	24	6(25 %)	18(75 %)	0.181
Age(years)				
> 55	56	20(36 %)	36(64 %)	
≦55	35	13(37 %)	22(63 %)	0.890
Smoking history				
Yes	54	20(37 %)	34(63 %)	
No	37	13(35 %)	24(65 %)	0.853
Pathological type				
Adenocarcinoma	54	18(34 %)	36(66 %)	
Squamous carcinoma	37	15(41 %)	22(59 %)	0.482
Tumor differentiation				
High	22	10(45 %)	22(55 %)	
Middle	58	21(36 %)	37(64 %)	
Low	11	2(18 %)	9(82 %)	0.307
Tumor location				
Right lung	45	17(38 %)	28(62 %)	
Left lung	46	16(35 %)	30(65 %)	0.766
Tumor size(cm)				
> 3	54	17(31 %)	27(69 %)	
≦3	37	16(43 %)	21(57 %)	0.252
Lymphatic metastasis				
Yes	42	8(19 %)	34(81 %)	
No	49	23(47 %)	26(53 %)	0.002
TNM classification				
I	43	22(51 %)	21(49 %)	
II	22	6(27 %)	16(73 %)	
III + IV	26	5(19 %)	21(81 %)	0.017

*Abbreviations*: *TNM* tumor-node-metastasis

^*^ χ^2^test

### MiR-361-3p inhibits NSCLC cell proliferation, migration and invasion in vitro

To functionally characterize miR-361-3p in NSCLC, we restored the expression of miR-361-3p by ectopic expression of this miRNA in the lowest expression NSCLC cell line (A549 and HTB-182) and downregulated miR-361-3p in the highest expression NSCLC cell line (SPC-A-1). To this end miR-361-3p stably overexpressed A549 ^miR-361-precursor^ and HTB-182 ^miR-361-precursor^ cells and stably downregulated in SPCA-1^anti-miR-361-3p^ cells were generated. The different levels of miR-361-3p expression were confirmed by real-time PCR (Fig. 1e).

**Fig. 2:**
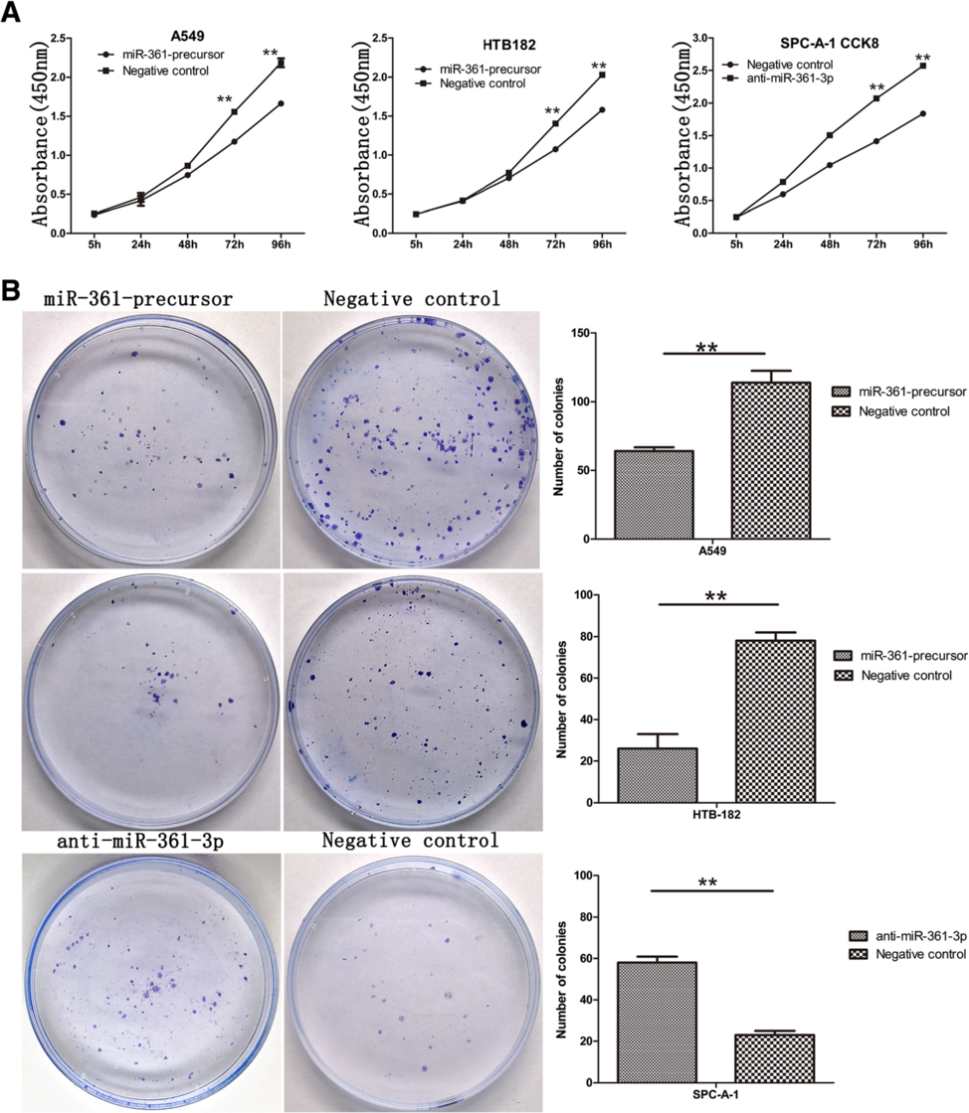
miR-361-3p inhibited NSCLC cells proliferation in vitro. **a** miR-361-3p inhibited NSCLC cells proliferation determined using CCK8 assays. **b** miR-361-3p inhibited NSCLC cells proliferation determined using colony formation. Data were represented as the mean ± SEM of three independent experiments. *P < 0.05, **P < 0.01

**Fig. 3:**
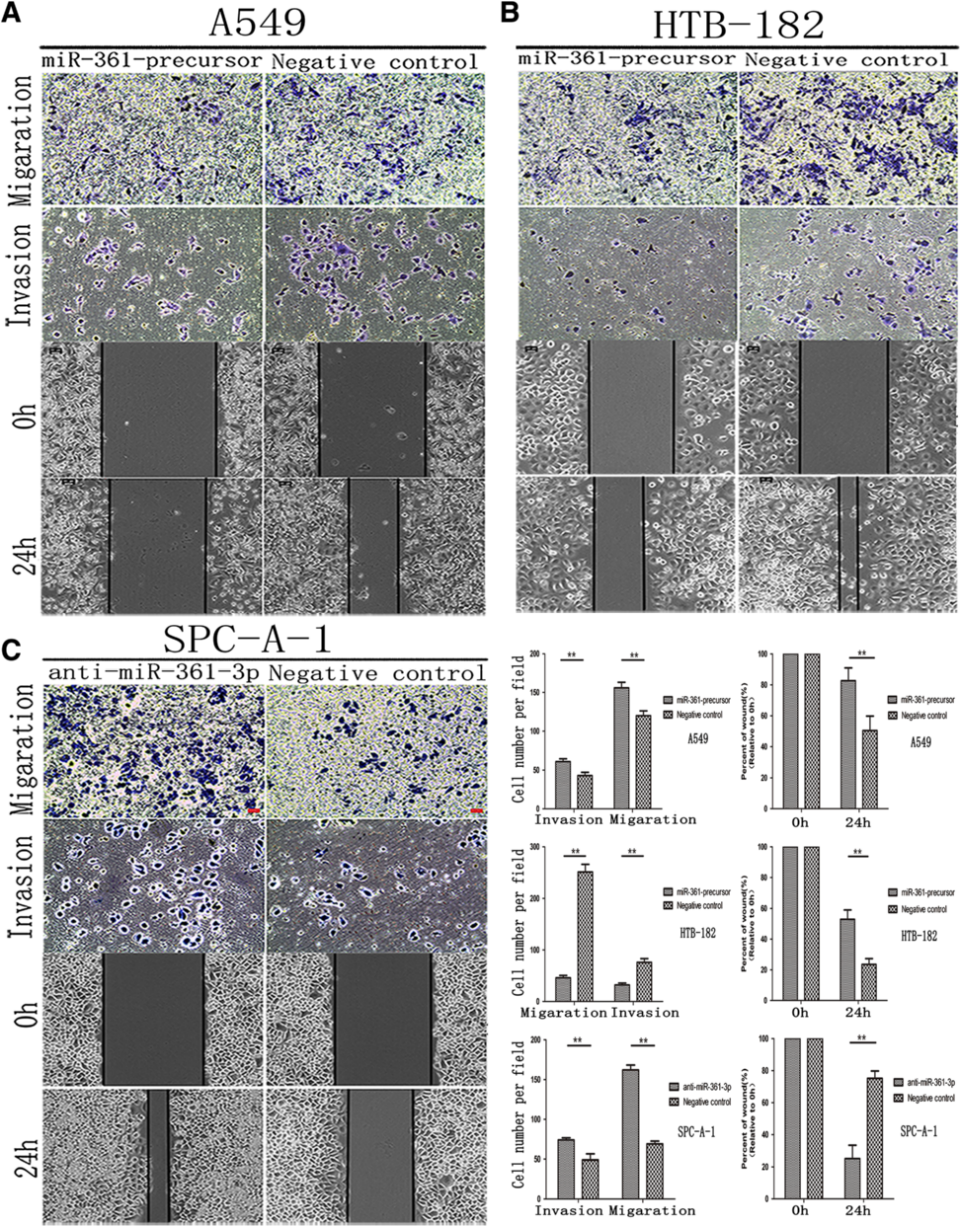
miR-361-3p inhibited NSCLC migration and invasion in vitro. **a** up-regulated miR-361-3p suppressed migration and invasion in vitro in A549. **b** up-regulated miR-361-3p suppressed migration and invasion in vitro in HTB-182. **c** down-regulated miR-361-3p enhanced migration and invasion in vitro in SPC-A-1 compared with controls. Data which were put beside picture **c** were represented as the mean ± SEM of three independent experiments. *P < 0.05, **P < 0.01

Cell Counting Kit-8(CCK8) and colony formation assays performed to assess the role of miR-361-3p in NSCLC cell proliferation. The result showed that compared with the control group, with forced expression of miR-361-3p impaired in both A549 ^miR-361-precursor^ and HTB-182 ^miR-361-precursor^ cells, Cell Counting Kit-8(CCK8) (Fig. 2a) and colony formation assays (Fig. 2b) showed that cell proliferation were significantly repressed. On the contrary, the cell proliferation of SPCA-1^anti-miR-361-3p^ cells was significantly enhanced. Similarly, wound healing assay showed wound closure of miR-361-3p impaired in both A549 ^miR-361-precursor^ and HTB-182 ^miR-361-precursor^ were significantly slow. Furthermore, transwell assays showed the migratory and invasive abilities was significantly decreased (Fig. 3a, b), whereas reduced miR-361-3p expression inSPC-A-1^anti-miR-361-3p^ cells resulted in the opposite result (Fig. 3c). These results indicated that miR-361-3p inhibits NSCLC cell proliferation and metastasis in vitro.

### Overexpression of miR-361-3p inhibits tumor growth and metastasis of NSCLC cells in vivo

**Fig 4:**
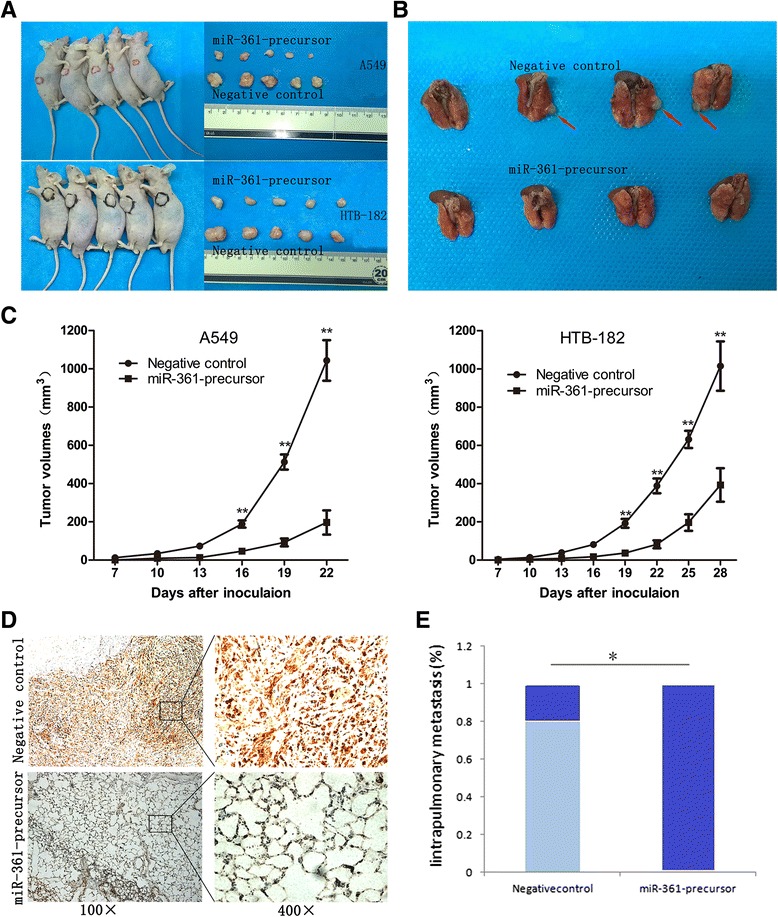
Overexpression of miR-361-3p inhibits NSCLC in vivo. **a** The NSCLC mouse subcutaneous implantation model in mice was constructed by using A549&HTB-182 cells infected with negative control and miR-361-precursor. Tumor volumes of subcutaneous implantation models of A549 are shown; and tumor volumes in the orthotopic implantation models at week 3or4 are shown. **b** Tumor volumes of intravenous metastasis models of A549 are shown. **c** Tumor growth curves of subcutaneous implantation models of NSCLC. **d** The expression of SH2B1 in local tumor tissues was determined by immunohistochemistry. **e** The percentage of mice with or without metastatic nodules in the lungs was calculated and compared. Data were represented as the mean ± SEM of three independent experiments. **P < 0.01

Given that miR-361-3p impaired the proliferation, migration and invasion of NSCLC cells in vitro, we examined whether miR-361-3p could affect tumorigenicity and metastasis in vivo. A549 and HTB-182 cells stably expressing miR-361-3p and negative control vector were injected subcutaneously into nude mice. Palpable tumors formed within 1 week. Tumor volume was measured each week, and mice were sacrificed 4 weeks after tumor cell implantation. The size of NSCLC tumors in these two groups was calculated and compared. The average tumor volume of A549 cells stably transfected with miR-361-precursor was significantly smaller than tumors in the negative control group (Fig. 4a). The tumor growth-curve of tumor volume was drawn according to time and a significant difference was shown between the two groups (Fig. 4c). To investigate the in vivo role for miR-361-3p in NSCLC cell migration, we examined the mice for lung metastasis of the A549 cells. As shown in Fig. 4, Immunohistochemistry confirmed that the expression of SH2B1 was significantly lower in A549^miR-361-precursor^ group than negative control groups (Fig. 4d). The intrapulmonary metastasis rate of A549 ^vector^ was 80 %, whereas no metastasis was found in A549 ^miR-361-precursor^ group (Fig. 4b, d). Together, these data support an important role formiR-361-3p in suppression of NSCLC growth and metastasis in vivo.

### SH2B1 is a direct downstream target of miR-361-3p

**Fig. 5:**
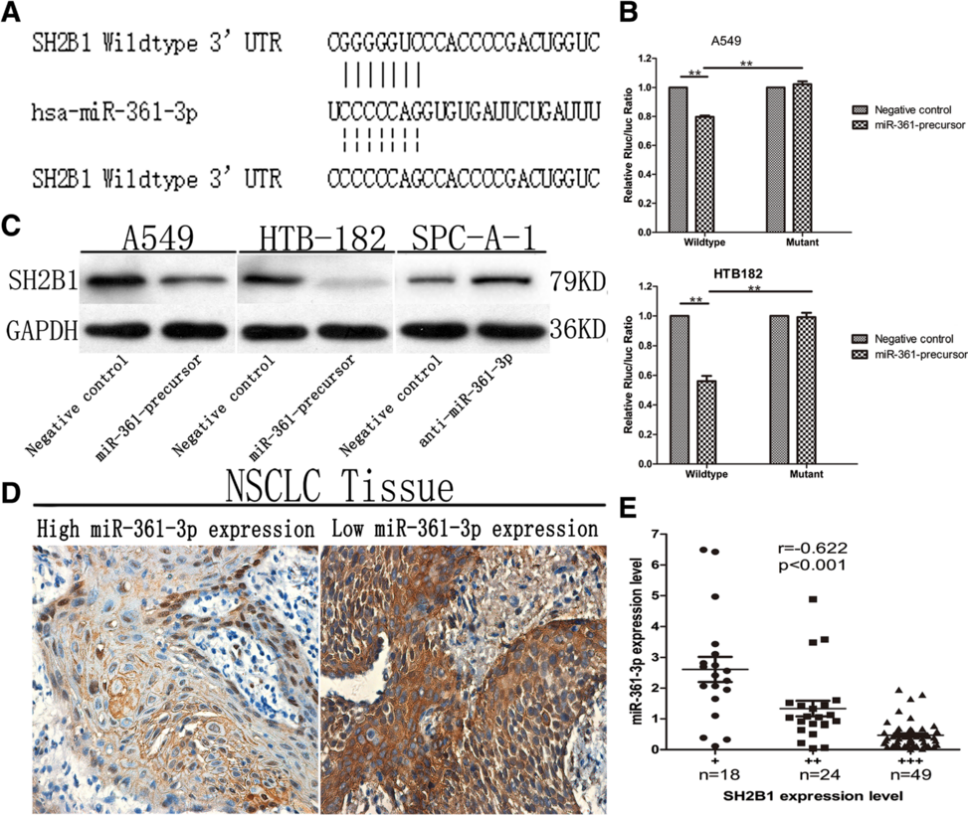
SH2B1 is a direct downstream target of miR-361-3p. **a** Schematic of the construction of wild-type or mutant SH2B1 3'-UTR vectors is illustrated. **b** Relative luciferase activity was analyzed in A549 and HTB-182 cells. Firefly luciferase vector was used as an internal control. **c** Western blot results of SH2B1 protein in A549, HTB-182 and SPC-A-1 cells infected with miR-361-precursor lentivirus or anti-miR-361-p. **d** The representative IHC pictures showed the SH2B1 protein expression in NSCLC tissue with high or low miR-361-3p expression. **e** The inverse correlation between the expression of miR-361-3p and SH2B1 in the same set of NSCLC tissue specimens. Data were represented as the mean ± SEM of three independent experiments. **P < 0.01

To explore the molecular mechanisms through which miR-361-3p regulates NSCLC cell proliferation and metastasis, we searched candidate target genes of miR-361-3p using publicly available databases. Among the candidates, SH2B1 exhibited one of the highest prediction scores and the most complementary structure with miR-361-3p (Fig. 5a). Moreover up-regulation of SH2B1 protein was found in various types cancer and high SH2B1 expression is associated with more aggressive phenotypes [18–20]. We then carried out a luciferase-based assay to validate whether these genes were indeed regulated by miR-361-3p. Luciferase vectors containing the 3’-UTR of each gene were created and transfected along with or without the miR-361-3p expressing plasmid into cells. Measurement of luciferase activity revealed that miR-361-3p expression was associated with marked reduction of the activity of SH2B1 UTR. The specificity of this inhibition was demonstrated by the finding that the activity of a mutant SH2B1 3’-UTR with the putative binding site mutated was not affected by miR-361-3p (Fig. 5b). In addition, western blot analysis showed that SH2B1 protein expression was clearly decreased in A549 cells and HTB-182 cells transfected with LV-miR-361-precursor, and increased in SPC-A-1 cells transfected with LV-anti-miR-361-3p (Fig. 5c). Furthermore, to explore the relationship between miR-361-3p and SH2B1 in clinical specimens, we examined SH2B1 expression using immunohistochemical analysis on FFPEs of 91 NSCLC specimens. SH2B1 expression was positively correlated with TNM stage and lymph node metastasis of NSCLC (*P* < 0.001) (Table 2). We compared SH2B1 expression data from immunohistochemistry analysis with results of miR-361-3p expression level from qRT-PCR analysis on specimens of these NSCLC tissues. There was an inverse correlation between miR-361-3p and SH2B1 expressions in these specimens (Fig. 5d, e, *R* = −0.622, *P* < 0.001). Taken together, these results indicated that SH2B1 was a direct downstream target for miR-361-3p in NSCLC cells.

**Table 2:** SH2B1 expression and clinicopathological features in non-small cell lung cancer (NSCLC) patients

Variables	n	SH2B1 expression			
		+	++	+++	*p* value^*^
Gender					
Male	67	16	16	35	
Female	24	3	6	15	0.486
Age(years)					
> 55	56	14	10	32	
≦55	35	5	12	18	0.156
Smoking history					
Yes	54	14	11	29	
No	37	5	11	21	0.293
Pathological type					
Adenocarcinoma	54	15	12	27	
Squamous carcinoma	37	4	10	23	0.063
Tumor differentiation					
High	22	7	6	9	
Middle	58	10	14	34	
Low	11	2	2	7	0.518
Tumor location					
Right lung	45	11	10	24	
Left lung	46	8	12	26	0.696
Tumor size(cm)					
> 3	54	8	12	34	
≦3	37	11	10	16	0.129
Lymphatic metastasis					
Yes	42	0	10	32	
No	49	19	12	18	<0.001
TNM classification					
I	43	18	10	15	
II	22	1	9	12	
III + IV	26	0	3	23	<0.001

*Abbreviations*: *TNM* tumor-node-metastasis

^*^ χ^2^test

### Gain- and loss-of-function of SH2B1 abrogated or mimicked impact of miR-361-3p on cell proliferation and metastasis

**Fig. 6:**
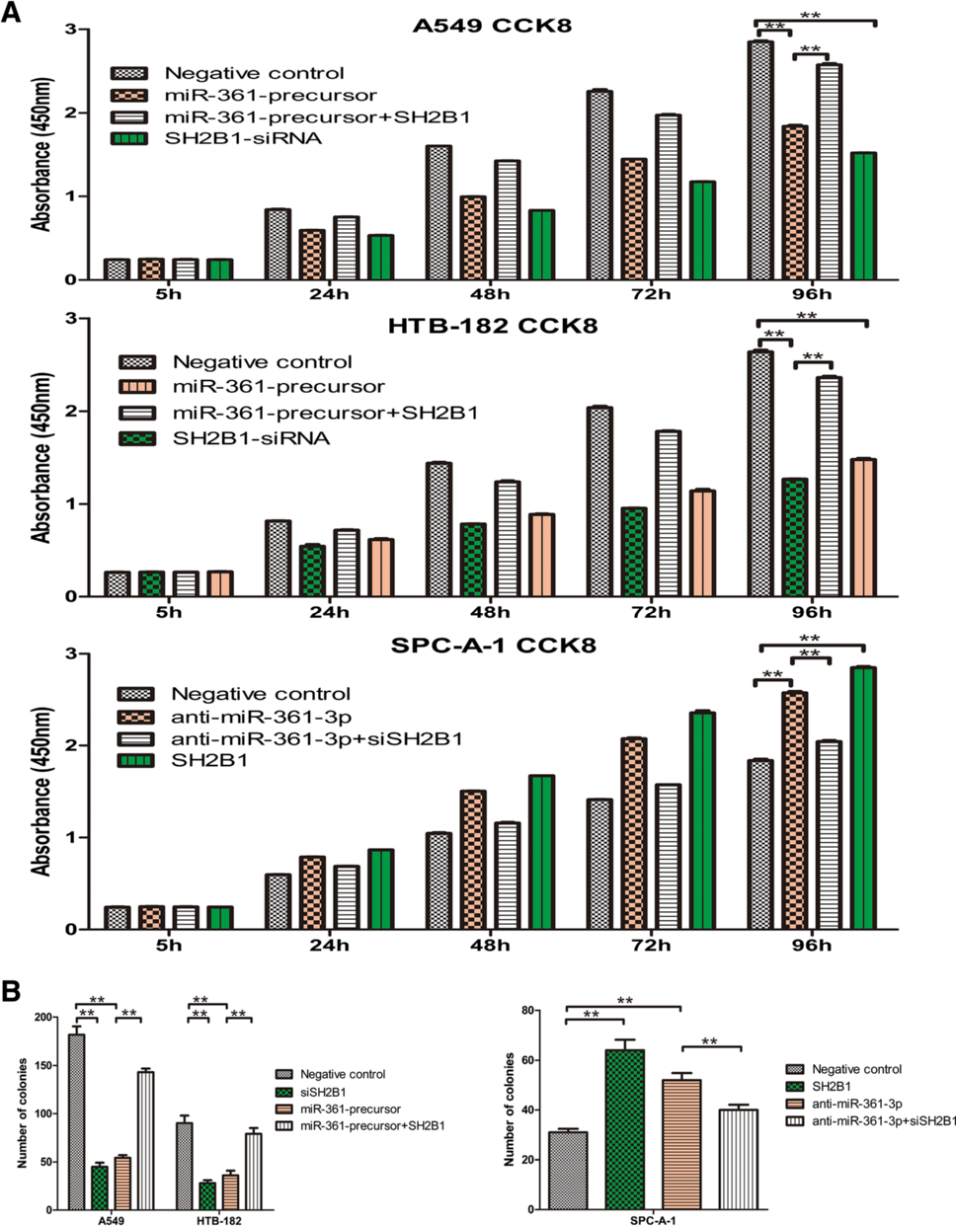
Gain- and loss-of-function study with SH2B1 expression vector and siSH2B1 in cells proliferation in vitro. To investigate whether SH2B1 expression may interfere or mimic the function of miR-361-3p, NSCLC cells were transfected by SH2B1 siRNA or SH2B1 expression vector to inhibit or restore the SH2B1 expression. CCK8 assay (**a**), colony formation (**b**). Data were represented as the mean ± SEM of three independent experiments. *P < 0.05, **P < 0.01

**Fig. 7:**
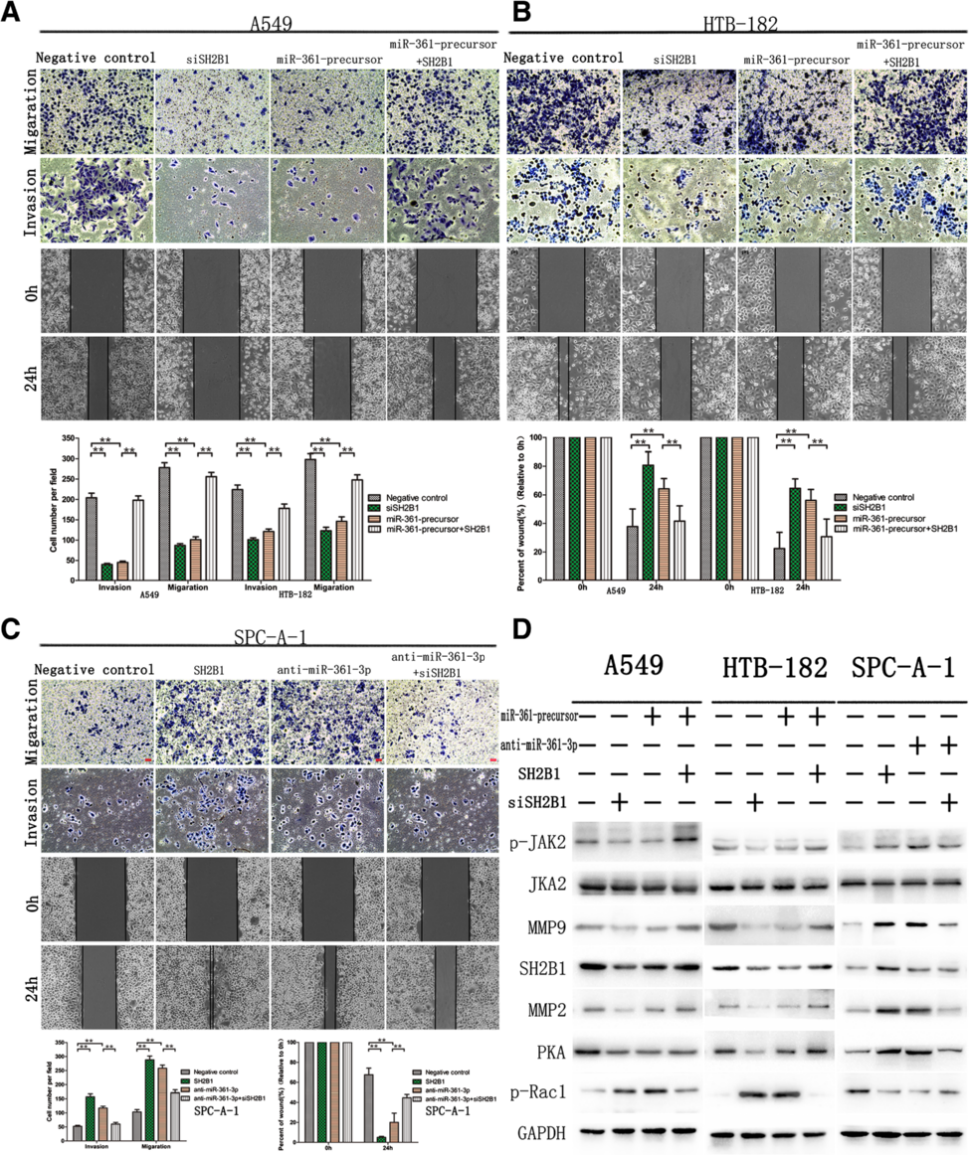
Gain- and loss-of-function study with SH2B1 expression vector and siSH2B1 in migration and invasion in vitro. **a** migration and invasion in vitro and wound healing assay in A549; **b** in HTB-182; **c** SPC-A-1. The date were put beyond. **d** Western blot of SH2B1 and its downstream proteins in stable infected NSCLC cells with SH2B1 expression, siSH2B1 or control vector reintroduced. Data were represented as the mean ± SEM of three independent experiments. *P < 0.05, **P < 0.01

To determine whether miR-361-3p-dependent inhibition of NSCLC cell proliferation and metastasis was indeed mediated by SH2B1, we used a complementary approach of gain- and loss-of-function of SH2B1. Specifically we transfected with si-SH2B1 to down the expression of SH2B1, and transfected with a SH2B1 expression vector to restore SH2B1 expression. The restoration of SH2B1 expression enhanced the proliferation (Fig. 6a) and colony formation (Fig. 6b) of A549^miR-361-precursor^ and HTB-182 ^miR-361-precursor^cells. Moreover, the restoration of SH2B1 significantly attenuated miR-361-3p-mediated inhibition of A549^miR-361-precursor^ and HTB-182 ^miR-361-precursor^ cells migration and invasion (Fig. 7a, b). In contrast, siSH2B1-mediated inhibition of SH2B1 expression mimicked the effect of miR-361-3p on proliferation and colony formation capacity in SPC-A-1 cell^anti-miR-361-3p^ (Figs. 6a, b and 7c).

We further investigated the role of SH2B1 in NSCLC progression. The results showed that SH2B1 overexpression significantly promoted wound healing of SPC-A-1cells (Fig. 7a). Downregulation of SH2B1 on the other hand inhibited the proliferation (Fig. 6a), cell migration and invasion of A549 (Fig. 7a) and HTB-182 cells (Fig. 7b). These results together support an oncogenic role of SH2B1 in NSCLC.

To explore whether miR-361-3p exerts its functions through the JAK2-SH2B1-Rac1 pathways that contribute to cancer proliferation, development and progression [20], we examined a number of the main SH2B1 signaling downstream target genes, including phosphorylation of Janus kinases 2(p-JAK2), phosphorylation of Rho GTPase Ras-related C3 botulinum toxin substrate 1(p-Rac1), cAMP-Protein Kinase Catalytic subunit (PKA), matrix metalloproteinase-2(MMP2), and MMP9. Overexpression of p-JAK2, PKA, MMP2 and MMP9 and downexpression of p-Rac1 were detected in A549 and HTB-182 cells that stably overexpressed miR-361-3p (Fig. 7d, lane 1–2). In contrast, downexpression of p-JAK2, PKA, MMP2 and MMP9 and overexpression of p-Rac1 were detected in SPC-A-1 cells with stable down-regulation of miR-361-3p SH2B1 exhibited lower SH2B1 expression (Fig. 7d, lane 3). These data indicate that miR-361-3p inhibits SH2B1 signaling in NSCLC, which involved tumor development and progression.

## Discussion

NSCLC is the most prevalent cancer types and has highest mortality rate in China [1]; however, the progression mechanisms of NSCLC have largely remained elusive. Ample evidence indicates a crucial role for miRNAs in human cancer [21], especially the miRNAs participate in the initiation, promotion, and progression of NSCLC. For instance, miR-21 promotes growth and invasion of NSCLC [22]. In addition, miR-494, miR-101, miR-1254, miR-574-5p, miR-143 and miR-181a were demonstrated to be involved in NSCLC [23–25]. In the present study, we certified that miR-361-3p was frequently down-regulated in NSCLC, and first found that the reduced miR-361-3p expression was closely related to advanced stage and lymph node metastasis of NSCLC. Furthermore, we demonstrated that overexpression of miR-361-3p could suppress NSCLC cell proliferation, migration and invasion in vitro and in vivo. The versatile functions of miR-361-3p in tumor cell proliferation, migration and invasion suggest its potential application as a prognostic predictor and cancer therapeutic target.

SH2B1, which is an Src homology 2 (SH2) and pleckstrin homology (PH) domain-containing protein, is known an adapter protein, and can bind the large number of kinases, such as Janus kinase (JAK)-2 and JAK1 [26]; fibroblast growth factor receptor-1 [27]; insulin receptor [28]; insulin receptor substrate-1 [29]. It was reported that SH2-Bβ functions as an adapter/scaffolding protein that recruits Rac and perhaps other proteins to activated membrane receptor-JAK complexes or receptor tyrosine kinases where they are then positioned appropriately to regulate the actin cytoskeleton and promote membrane ruffling and cell motility [20]. In our study, we demonstrated that miR-361-3p can bind to a sequence within the 3’-UTR of SH2B1 by luciferase-based reporter assay. MiR-361-3p-mediated control of SH2B1 expression was further validated by complementary gain- and loss-of-function approaches. Importantly, ectopic SH2B1 expression could effectively impede the ability of miR-361-3p to inhibit proliferation and metastasis. Moreover, knockdown expression of SH2B1 abrogated the effects induced by miR-361-3p-inhibitor. Our study provides solid evidence to support that miR-361-3p inhibit proliferation and metastasis of NSCLC by directly targeting SH2B1. For further study, we examined the expression of SH2B1 signaling downstream target genes and found that expression of p-JAK2, PKA, MMP2 and MMP9 were decreased and p-Rac1 was increased in NSCLC cells that stably overexpressed miR-361-3p. In contrast, expression of p-JAK2, PKA, MMP2 and MMP9 was significantly up-regulated and p-Rac1was down-regulated in NSCLC cells that stably expressed miR-361-3p inhibitor. This suggests that SH2B1 activation resulted in increasing migration and invasion is likely through activation of JAK2/Rac1 that regulates cell morphology and mobility as well as membrane trafficking. Rac1 is a member of the Rho family of small GTPases and participates in numerous pathways inducing cytoskeleton reorganization, gene transcription, cell proliferation and survival [30, 31], especially in lung cancer modifies lung cancer migration, invasion and actin cytoskeleton rearrangements and enhances chemosensitivity to antitumor drugs [32, 33]. Schwarz J et al. reported that phosphorylation of Rac1 at serine-71 affects Rac1 activity by shift specificity of GTPase/effector coupling and modulates downstream signaling [34]. Thus, down-regulation of p-Rac1 through inhibition of SH2B1 could be a mechanism by which miR-361-3p suppresses cell proliferation, migration and invasion. MMPs are a family of enzymes that proteolytically degrade various components of the extracellular matrix [35]. High levels of certain MMPs are closely correlated with the invasive and metastatic potential of tumors [36, 37]. Specifically, activated Rac1 regulates tumor invasion of lung cancer cells by regulating gene transcription of MMP2 and MMP9 [38]. These data indicate that miR-361-3p suppresses progression of NSCLC through inhibition of the versatile tumor-promoting SH2B1. It is noteworthy that neither miR-361-3p nor SH2B1 has been investigated in NSCLC. Our report revealed a novel miR-361-SH2B1 axis in regulation of NSCLC.

SH2B1 is identified as a target of miR-361-3p, but SH2B1 might not solely be explained the antioncogenic properties of miR-361-3p, because a single miRNA can potentially regulate dozens to hundreds of genes in tumorigenesis [39]. Therefore, future studies to identify additional novel targets of miR-361-3p and other miRNAs that can also regulate SH2B1 will allow us to have deep understanding of the mechanisms underlying the development and progression of NSCLC.

In conclusion, our results show that miR-361-3p is significantly downregulated in NSCLC. This miRNA can potently inhibit NSCLC cell proliferation and metastasis in vitro and in vivo. The tumor-suppressive role of miR-361-3p is largely mediated by one of its target, Sh2B1. These findings provide new insight into the molecular pathogenesis of NSCLC and implicate miR-361-3p as a potential prognostic biomarker and therapeutic target of NSCLC.

## Conclusions

Carcinogenesis is a series of sequential events, including growth, proliferation, migration, and local invasion. Herein, we showed that miR-361-3p could suppress the carcinogenesis of NSCLC through inhibition of growth, proliferation, migration and invasion. Furthermore, our evidence suggests that miR-361-3p is a potential therapeutic target in NSCLC. Further studies are required to fully understand the detailed mechanisms of miR-361-3p in NSCLC carcinogenesis and as a potential therapeutic approach.

## Methods

### Ethical statement

Written informed consent was obtained from all participants, and the study protocol was approved by the ethics committee of Xiangtan Hospital, Central South University (CSU). All mouse experiments were approved by the Animal Care and Use Committee and conducted in accordance with the official recommendations of the Care and Use Laboratory Animals of Xiangtan Hospital, CSU.

### Patient and tissue samples

Primary cancer tissues and paired adjacent non-tumor tissues were collected from 91 patients with NSCLC underwent lung resection at the Department of Surgery, Xiangtan Hospital of Central South University from March 2013 to June 2014. Patients did not receive any preoperative cancer treatments, such as radiotherapy or chemotherapy. Each specimen was rapidly frozen in liquid nitrogen, and transferred to the −80 °C refrigerator for subsequent experiments. The collected samples were confirmed by an experienced pathologist. The clinical data of NSCLC patients including tumor-node metastasis (TNM) staging were also collected.

### Cell lines and cell culture

Six NSCLC cell lines (HTB-182, A549, SPC-A-1, H1299, PC-9, LTEP-A-2) were obtained from the American Type Culture Collection. A normal human bronchial epithelial cell line (HBE), were purchased from the Institute of Biochemistry and Cell Biology of the Chinese Academy of Sciences (Shanghai, China). Cells were cultured in RPMI 1640 (GIBCO-BRL) medium supplemented with 10 % fetal bovine serum (10 % FBS), 100 U/ml penicillin, and 100 μg/ml streptomycin (Biyuntian, China) in humidified air at 37 °C with 5 % CO2.

### RNA extraction and qRT-PCR analyses

Total RNA was extracted from cell lines and frozen tumor specimens using Trizol reagent (Invitrogen, Carlsbad, CA, USA) according to the manufacturer’s protocol. The qRT-PCR assays were performed to detect miR-361-3p and SH2B1 expression using the PrimeScript RT reagent Kit and SYBR Premix Ex Taq (GeneCopoeia, USA) according to the manufacturer’s instructions. The relative level of miR-361-3p and SH2B1 was determined by qRT-PCR using gene specific primers. U6 or β-actin was used as a normalization control. Levels of miR-361-3p and SH2B1 were normalized to U6 and β-actin, respectively, to yield a 2^-ΔΔCt^ value for relative expression of each transcript. Experiments were repeated at least three times. The RT reaction was carried out under the following conditions: 37 °C for 60 min; 85 °C for 5 min; and then held on 4 °C. After the RT reaction, the complementary DNA products were diluted at 1:5 and 2 μl of the diluted complementary DNA was used for subsequent qRT-PCR reactions. The qRT-PCR primers were designed as follows: miR-361-3p, Forward: 5′-UCCCCCAGGUGUGAUUCUGAUUU-3′, Reverse: 5′-GCAAATCAGAATCACACCTG-3′. U6, Forward: 5′-CTCGCTTCGGCAGCACA-3′, Reverse: 5′-AACGCTTCACGAATTTGCGT-3′; Human SH2B1, Forward: 5′-GACAACCACAGCCCTGGAGAT-3′, Reverse: 5′-AGACACCCAGGCCTTCACAT-3′. Human β-actin, Forward: 5′-GCACCACACCTTCTACAATGAG-3′, Reverse: 5′-GATAGCACAGCCTGGATAGCA-3′. Human GAPDH, Forward: 5′- TGCACCACCAACTGCTTAGC-3′, Reverse: 5′-GGCATGGACTGTGGTCATGAG-3′. The qRT-PCR reaction was conducted at 95 °C for 10 min and followed by 40 cycles of 95 °C for 10s, 60 °C for 30 s and 72 °C for 30 s in the ABI 7500 real-time PCR system (Applied Biosystems, CA, USA). The qRT-PCR results were analyzed and expressed as relative miRNA expression of CT (threshold cycle) value, which was then converted to fold changes.

### Vector construction and transfection

The hsa-mir-361-precursor sequence was constructed as follows: (Forward) hsa-miR-361-Age I-F GAGGATCCCCGGGTACCGGTGCAGTGGCACGCTTGACAGTGATTTTTTTCCTGGGATTTGGGAGC, (Reverse) hsa-miR-361-Nhe I-R CACACATTCCACAGGCTAGTAGGGAGCTCAACCATACCAG. The sequence was amplified and cloned into the pGC-FU-3FLAG-SV40-EGFP Vector (GeneChemCo., Shanghai, China) to generate pGC-FU-miR-361 and the pGC-FU-3FLAG-SV40-EGFP Vector only as negative control. The hsa-miR-361-3p-inhibition sequence was constructed as follows: (Forward) hsa-miR-anti-361-3p-AgeI-F AATTCAAAAATCCCCCAGGTGTGATTCTGATTT, (Reverse) hsa-miR-anti-361-3p -EcoRI-R CCGGAAATCAGAATCACACCTGGGGGATTTTTG. The sequence was amplified and cloned into the pGCSIL-008 Vector to generate pGCSIL-008-miR-anti-361-3p. The non-silencing shRNA control sequences (TTCTCCGAACGTGTCACGT) was cloned into the pGCSIL-008 Vector as negative control (pGCSIL-008-RNAi-NC-LV). The SH2B1 expression vector was constructed by inserting its CDS sequence into the pEX-2 vector (GeneChem, Shanghai, China). Virus packaging, production and cell transfection were performed according to the manufacture’s protocol. The expression was validated by qRT-PCR. SH2B1-siRNA (si-SH2B1) and non-specific control siRNA (si-NC) were purchased from GeneChem, Shanghai, China.

### Cell proliferation and colony formation assays

Cell proliferation was monitored using CCK8 (Sigma). LV-miR-361-precursor, LV-negative control transfected A549 and HTB-182 or anti-miR-361-3p, or pGC FU-RNAi-NC-LV (Negative control) transfected SPC-A-1 cells (3000 cells/well, 5 wells/group) were allowed to grow in 96-well plates. Cell proliferation was documented every 24 h following the manufacturer’s protocol. CCK-8 reagent was added to each well at 1.5 h before the endpoint of incubation. The optical density (OD) 450 nm values were determined by a microplate reader. All experiments were repeated at least three times. For the colony formation assay, LV-miR-361-precursor, LV-negative control transfected A549 and HTB-182 cells or LV-anti-miR-361-3p, pGC FU-RNAi-NC-LV (Negative control) transfected SPC-A-1 cells (100/well) were allowed to grow in culture dish (8 cm^2^) and maintained in media containing 10 % FBS, replacing the medium every 4 days. After 14 days, cells were fixed with methanol and stained with 10 % Giemsa (Solarbio, Beijing, China). Only positive colonies (diameter > 40 um) in the dishes were counted and compared [40]. All experiments were performed in triplicate.

### In vitro cell migration and invasion assays

For the migration assays, 48 h after transfection, 2 × 10^4^ cells in serum-free media were placed into the upper chamber of an insert (8 μm pore size, BD). For the invasion assays, 4 × 10^4^ cells in serum-free media were placed into the upper chamber of an insert coated with Matrigel (BD, USA). Media containing 10 % FBS were added to the lower chamber. For migration assays, after 24 h of incubation, and for invasion assays, after 24 h of incubation, removing the cells remaining on the upper membrane with cotton wool, whereas the cells that had migrated or invaded through the membrane were stained with 10 % Giemsa in methanol, imaged, and counted using an inverted microscope (Canon, Japan). For wound-healing assay, cells (1 × 10^6^ cells) were seeded in six-well plates, cultured overnight and transfected with miR-361-precursor, negative control or anti-miR-361-3p, pGC FU-RNAi-NC-LV (Negative control). Upon reaching the 95–100 % confluence, the cell layer was scratched with a 10 μl pipette tip and washed with culture medium twice and cultured again for up to 24 h with serum-free medium. Images were captured at different time points (0, 24 h) and the same areas under a microscope to assess the rate of gap closure. The wound width of 6 random views was measured, and the healing width was calculated by wound width at 0 h time point minus wound width at 24 h time point and normalized by solvent control [41]. Every experiment was repeated three times. Bioinformatics methods using bioinformatics software (DIANA TOOL, Targetscan, miRanda) to predict miR-361-3p potential target gene, combined with the literature and through the test screening, SH2B1 was selected as a further object of study.

### Luciferase reporter assay

To construct a luciferase reporter vector, SH2B1 3′-UTR fragment containing putative binding sites for miR-361-3p was amplified by PCR using the following primers: h-SH2B1-F: GCGCTCGAGCTATCCAGAACCGACCACC h-SH2B1-R:AATGCGGCCGCCACGATAGAACCGAGATAA, the PCR product was subcloned downstream of the luciferase gene in the pLUC Luciferase vector (Ruibo, Guangzhou,China) and named SH2B1-3′-UTRWT.For the mutated construct, using the following primers: h-SH2B1-mut-F:GCCCCACCGACCCCCCCATTTCCCCATTAACTA. h-SH2B1-mut-R: GGAAATGGGGGGGTCGGTGGGGCTGACCAGAAG.A549^miR-361-precursor^ and HTB-182^miR-361-precursor^ cells grown in 96-well plate were transfected with 100 ng of SH2B1-3′UTR-Wt or SH2B1-3′UTR-Mut, using the Lipofectamie 3000 (Invitrogen, USA). After 72 h of transfection, luciferase activity was assessed according to the Dual-Luciferase Reporter Assay protocol (Promega, Madison, WI). Each experiment was repeated in triplicates.

### Western blotting

Total protein was extracted by lysing cells in RIPA buffer containing protease inhibitor. Protein samples were separated by sodium dodecyl sulfate polyacrylamide gelelectrophoresis (SDS-PAGE) and transferred onto polyvinylidenefluoride (PVDF) membranes. After blocking with 5 % non-fat milk or 3 % BSA in TBS-T, membranes were incubated with the primary antibody. The following antibodies were used: SH2B1 (1:1000,Abcam, USA), JAK2 (1:600, Abcam, USA), p-JAK2 (1:2000, Abcam, USA), p-Rac1 (1:500, Abcam, USA), Anti-cAMP Protein Kinase Catalytic subunit (1:60000,Abcam,USA),MMP2 (1:2000,Abcam.,USA), MMP9 (1:1000, Abcam,USA), GAPDH (1:10000, Abcam, USA) and goat-anti-rabbit IgG conjugated to horseradish peroxidase (HRP) (1:5000, Santa Cruz, USA),which was used as the secondary antibody. Cells were seeded on 10 cm cell culture plates, grown to 80 % confluences, and serum starved overnight. Target signals were quantified by BandScan software (Bio-Rad, Hercules, CA) and defined as the ratio of target protein relative to GAPDH.

### NSCLC mouse model

Five-week-old BALB/C-nu nude male mice were used for animal studies, and all animals were maintained in the specific pathogen-free (SPF) conditions at our institution. For the in vivo tumor proliferation assay, 3 × 10^6^ A549 cells transfected with LV-miR-361-precursor or LV-negative control were injected subcutaneously into the nude mice (5 per group). Tumor growth was monitored by caliper measurement once or twice a week for at least 4 weeks. Tumor volume was calculated as follows: V = L× l^2^ × 0.5, where L and l represent the larger and the smaller tumor diameters, respectively. The mice were sacrificed after 4 weeks. For the in vivo tumor metastasis assay, 2 × 10^6^ A549 cells transfected with LV-miR-361-precursor or LV-negative control were injected by the tail vein into the nude mice (5 per group). The mice were sacrificed after 2 months. At the time of killing the lung were removed and immediately fixed in 4 % (w/v) paraformaldehyde overnight and treated for immunohistochemistry.

### Immunohistochemical staining

Formalin-fixed, paraffin-embedded tissues were cut into 4-μm sections. Following deparaffinization, sections were rehydrated and subjected to antigen retrieval by microwaving in 0.01 M sodium citrate (pH 6) for 10 min. Sections were incubated at 4 °C overnight with monoclonal antibodies against SH2B1 as mentioned above. Immunostaining was performed using ChemMate DAKO EnVision Detection Kit, Peroxidase/DAB, Rabbit/Mouse (code K5007, DakoCytomation, Glostrup, Denmark) according to the manufacturer’s instructions [42]. Subsequently, sections were counterstained with hematoxylin (Dako) and mounted in dimethyl benzene. Protein staining was evaluated under a light microscope at 100 × and 400 × magnification. Staining intensity was scored manually by two independent experienced pathologists as 0 = no staining, 1 = weak staining, 2 = moderate staining, and 3 = strong staining. Tumor cells in five fields were randomly selected and scored based on the percentage of positively stained cells (0–100 %), as follows: 0, less than 5 %; 1, 5 to 25 %; 2, 25 to 50 %; or 3, more than 50 %. The final ICH score was then calculated by adding the two above scores, and scores of 0–2 were considered as low expressions while scores of 3–6 were defined as high expressions [43, 44].

### Statistical analysis

The relationship between miR-361-3p expression and clinicopathologic parameters was analyzed using the Pearson *χ*^2^ test. Spearman’s correlation analysis was used to determine correlation between miR-361-3p and SH2B1 expression. The differences between groups were analyzed using Student *t* test when there were only two groups, or assessed by one-way ANOVA when there were more than two groups. All statistical analyses were performed using the SPSS software (version 19.0, Chicago, IL). A two-tailed value of *P* < 0.05 was considered statistically significant.
